# The Impact of Bundled Payment Initiatives in Orthopedic Surgery Access to Care: Cherry Picking and Lemon Dropping

**DOI:** 10.7759/cureus.57205

**Published:** 2024-03-29

**Authors:** Zachary C Lum

**Affiliations:** 1 Orthopedic Surgery, Nova Southeastern University, Fort Lauderdale, USA

**Keywords:** comprehensive cost for joint replacement, bundled payment for care improvement, lemon dropping, cherry picking, total hip arthroplasty, total knee arthroplasty

## Abstract

This editorial explores the impact of bundled payment initiatives, specifically in the context of orthopedic surgery, on access to care. We examine the phenomenon of "cherry picking" healthier patients and "lemon dropping" higher-risk patients, potentially leading to disparities in access and healthcare outcomes. We discuss recent studies investigating these concerns and highlight the need for more in-depth research to better understand the groups these policies may marginalize. Policymakers are urged to consider measures to protect disadvantaged patients and ensure equitable access to care, aligning with the principles of equality and diversity in healthcare.

## Editorial

It is a well-known fact that the Centers for Medicare/Medicaid Services (CMS) is charged with the task of reducing overall spending in healthcare, as it is increasing to levels that eventually may become unsustainable. This usually results in dramatic spending reductions that affect all pillars of the healthcare pathway, and unfortunately will most affect the disadvantaged patient. One way CMS is attempting to control costs is to introduce bundled cost initiatives called Bundled Payments for Care Improvement (BPCI) and Comprehensive Care for Joint Replacement (CJR). These pay the healthcare institutions, organizations, or hospitals a single payment for a 90-day episode of care. These episodes of care include postoperative rehabilitation, whether in a rehabilitation facility or in an outpatient facility, and any potential readmissions or reoperations. Complications and reoperations can result in monetary loss for the healthcare institution, and thus there is motivation to avoid these. Organizations that perform well in certain metrics like lower readmissions or reoperations may receive additional payments as an incentive to participate in this program. This can result in organizations being financially incentivized to provide better care to lower their patient-related complications.

The potential drawback to this model is that patients who have a known higher risk for complications, readmissions, or reoperations may not be offered surgery, thus resulting in a loss of access to appropriate care [1-2]. Oftentimes, the patients affected with social determinants of health fall into these categories associated with diabetes, congestive heart failure, stroke, and obesity. Additionally, patients with lower risk, i.e., healthier, may be offered surgery more often than their other counterparts. There is evidence in many studies revealing Black, Asian, and Hispanic populations underwent lower rates of total knee arthroplasty (TKA) compared to their White counterparts [3-4].

Controversy exists between the bundled payment models and whether both an access to care issue is occurring and a selection bias that may result in poor cost-effectiveness of these models. This phenomenon may occur due to “cherry picking”, where patients who are healthier are selected for ambulatory surgery centers where healthcare organizations may not be participating in the bundled payment, and “lemon dropping”, where higher-risk patients are being done in inpatient settings resulting in higher costs or not being offered surgery at all, resulting in a denial of access to care.

Although it is a poor statement to say that more research is needed, I believe a better term would be that more in-depth research is required. Two studies investigating such concerns of “cherry picking” and “lemon dropping” were published recently. The first, by Humbyrd et al. [5], investigated multiple factors including age, race, gender, and ethnicity between pre-policy and post-policy implementation of the CJR model. While they could not find a significant difference, they only measured the span of a year before and after the implementation of the policy. Subtle effects such as these, usually are not effective within a year timespan. Logistically speaking, planning to undergo TKA can become a months-long process, meaning that patients who may have been scheduled for knee or hip replacement had already been selected during the pre-policy time period and underwent the surgery in the post-policy timeframe. A better way to look for disparities would be to investigate the trends over time. 

The other study by Bernstein et al. [6] was a systematic review investigating differences in sociodemographics, comorbidities, case complexity, and healthcare usage in 10 studies reporting on bundled payment initiatives. They found small changes in six of the 10 studies of lower orthopedic surgery utilization in patients with sociodemographic differences; one study revealed a lower number of treatments for patients with disabilities, and two revealed lower surgical utilization in patients who were at prior acute or long-term care facilities. While these were small changes, they were still evident. Additionally, these policy changes were only monitored over a short time span of three years. As we know, policy changes that have a significant effect usually occur insidiously and as such need long-term trending. Lastly, these effects were measured only for patients who underwent surgery. It did not consider patients who were not offered an operation. 

Two final factors that were not considered in this were the implications of the change of procedures from the inpatient-only list to outpatient, which resulted in a shift of over 50% of patients being placed into outpatient surgery, and the effects of the pandemic, which have both shifted younger and healthier patients away from these bundled initiatives controlled by healthcare organizations to ambulatory surgery centers (ASCs) where these patients may not be captured [7-8]. Typically younger patients are more prevalent in ASCs, which usually result in lower complication rates. The shift of patients from these bundled initiatives to non-captured data does not factor this into consideration.

To conclude, studies that have investigated the trends of pre-policy and post-policy implementation need to be conducted in order to better understand the groups that these policies can marginalize. Better yet, policymakers should consider better protecting those groups of patients who are most disadvantaged by their policies and ensure that, as a nation of immigrants, the principles of equality, diversity, and equitable access to healthcare are upheld and preserved for all.
